# Developing Eco-Friendly and Cost-Effective Porous Adsorbent for Carbon Dioxide Capture

**DOI:** 10.3390/molecules26071962

**Published:** 2021-03-31

**Authors:** Mahboubeh Nabavinia, Baishali Kanjilal, Noahiro Fujinuma, Amos Mugweru, Iman Noshadi

**Affiliations:** 1 Department of Chemical Engineering, Rowan University, Glassboro, NJ 08028, USA; nabavinim1@students.rowan.edu (M.N.); fujinuma@rowan.edu (N.F.); 2 Department of Bioengineering, University of California, Riverside, CA 92516, USA; baishali.kanjilal@gmail.com; 3 Department of Chemistry, Rowan University, Glassboro, NJ 08028, USA; mugweru@rowan.edu

**Keywords:** carbon dioxide, capture, mesoporous polymer, cobalt, nickel

## Abstract

To address the issue of global warming and climate change issues, recent research efforts have highlighted opportunities for capturing and electrochemically converting carbon dioxide (CO_2_). Despite metal doped polymers receiving widespread attention in this respect, the structures hitherto reported lack in ease of synthesis with scale up feasibility. In this study, a series of mesoporous metal-doped polymers (MRFs) with tunable metal functionality and hierarchical porosity were successfully synthesized using a one-step copolymerization of resorcinol and formaldehyde with Polyethyleneimine (PEI) under solvothermal conditions. The effect of PEI and metal doping concentrations were observed on physical properties and adsorption results. The results confirmed the role of PEI on the mesoporosity of the polymer networks and high surface area in addition to enhanced CO_2_ capture capacity. The resulting Cobalt doped material shows excellent thermal stability and promising CO_2_ capture performance, with equilibrium adsorption of 2.3 mmol CO_2_/g at 0 °C and 1 bar for at a surface area 675.62 m^2^/g. This mesoporous polymer, with its ease of synthesis is a promising candidate for promising for CO_2_ capture and possible subsequent electrochemical conversion.

## 1. Introduction

With a significant rise in the average atmospheric CO_2_ concentration from levels in the preindustrial age, deleterious effects on global warming and climate change have become visible [1,2,3]. Consequently, environmental concerns have convinced international bodies and governments to take steps to address policies pertaining to CO_2_ emissions. Some of the most remarkable strategies are CO_2_ capturing and conversion technologies, such as chemical, photocatalytic [4,5], and electrocatalytic reduction [6,7,8], for which the first step is efficient CO_2_ capture. In many of these technologies, heterogenous conversion catalysts, function [9,10,11], in the first step as, solid adsorbents and some of these entail materials such as silica [12,13,14], metal-organic frame (MOFs) [3], and carbon-based materials [14,15,16,17]. Some commonly researched carbon-based adsorbent materials entail activated carbon [18], ordered mesoporous polymer/carbon (OMP/OMC) [14,19,20], activated carbon fibers [21,22], graphene [2,17], and graphene oxide [23,24,25]. Regular pore structure, high surface area, and inexpensive precursors proffer potential advantages in OMP/OMC for CO_2_ capture [14,19,20]. The primary bottleneck in designing a feasible and cost-effective carbon capture and conversion process lies in producing a highly efficient carbo capture porous structure which also proffers feasibility of eco-friendly process scale-up along with high capture capacity, in addition to being retrofittable to sustainably convert CO_2_ to chemicals and fuels [1,26,27]. In several works, adsorption capacity and activity of adsorbents has been enhanced using functionalization of the catalyst surface and pore lining [14,20,28]. The basicity of nitrogenous functional groups, such as amines, provide sites for enhanced interactions with acidic CO_2._ Thus, doping via functionalization with basic nitrogen containing groups is considered as the most attractive methods to capture CO_2_ [22,26,29]. Fujian et al. showed that nitrogen not only increased CO_2_ capture but also made anchoring metal elements to polymer network feasible [20]. They synthesized an ordered mesoporous phenol- formaldehyde polymer by using hexamethylenetetramine (HMTA) as a source of nitrogen. These structures exhibited high stability, CO_2_ capture capacity and selectivity [20]. The anchoring of a transition metal to this framework also makes it probable to speculate its possible application as a CO_2_ conversion catalyst, provided a high enough CO_2_ capture capacity can be ensured.

Polyethylenimine (PEI) is a multipurpose polymer with amine-containing repeat units, spaced out by the aliphatic ethylene CH_2_CH_2_ ligand [30]. Due to its high basicity, high amine content, good thermal stability, and low volatility it can be used to produce highly effective and stable functionalized adsorbents [31,32]. Silica [33,34], nanocarbon tube, ordered mesoporous carbon, polymer [31,35] and alumina [36] have been studied with PEI modifications. PEI functionalized Polymethyl methacrylate (PMMA), and polystyrene (PS) [35] showed high CO_2_ adsorption capacity. Even though these structures have shown high CO_2_ capture capacities their commercial application has not been realized yet. The main hindrance to their commercial application is complicated, multi-stage synthesis method. This not only increases the cost of adsorbent, but also puts significant limitations on the scalability [31,37]. To overcome this, Wang et al. have developed organic amine- mediated synthesis of polymers for CO_2_ capturing and energy storage [38].

Solid metal-doped polymers are considered effective and environment-friendly catalyst for carbon dioxide conversion. Their advantageous features include high surface area, thermal stability, and good activity [2]. MOFs have also been reported as good adsorbents and catalysts owing to the active metal sites [3]. Cobalt, zinc, and nickel have been extensively used for this purpose. In addition to enhanced CO_2_ capture, these metals also facilitate photo or chemical-electro conversion. The solvothermal method is a common method for metal impregnation into heterogeneous structures which these compounds use to modify organic frame for improving capture and entrapment of CO_2_ and its possible subsequent conversion [2,3]. The elimination of multistep catalyst synthesis can pave way to realizable scale-up and hence commercial application.

This paper focuses on the potential of nitrogen-doped mesoporous polymer (MRF), made using PEI, with and without a metal center. The MRFs have been studied for CO_2_ capturing for possibility subsequent electrochemical conversion. In this paper, we studied cobalt and nickel doped MR. The effect of metal and PEI concentration on MRF properties such as surface area, pore size, pore structures and morphology, thermal stability were studied. For the synthetic procedure we used resorcinol and formaldehyde as precursors. Contrary to multi-stage synthesis, the one-pot solvothermal method not only decreases the cost of production by practically eliminating the use of high solvent volumes, it also eliminates scale- up barriers. In addition, low temperature of synthesis without the calcination stage makes it an ecofriendly process. The MRFs exhibit superior surface area and a uniform mesoporous structure, as observed from scanning electron microscopy. The elemental analysis map confirmed homogenous metal distribution. This approach also provides the opportunity for producing multi metal centered catalyst structures.

## 2. Results

### 2.1. Chemical Structure and Thermal Stability

The results of elemental analysis, on various compositions, is presented in Table 1. EDS elemental analysis of these compositions is illustrated in Supplementary Materials Figures S1 and S2. Elemental mapping results, as shown in Table 1, confirm that an increase in PEI concentration corresponded to an increase nitrogen content. Figures S1 and S2 underscore uniformity of distribution of cobalt and nickel in the test sample. PEI plays an important role in developing a uniform mesoporous structure. Nitrogen is indicative of the presence of the amine group of the branched PEI structure. The presence of nitrogen helps to increase nanoporosity by tethering to the Co or Ni nucleation centers while participating in the crosslinking kinetics on the other. While the nitrogen content in H-RF is expected to be double that of M-RF, it may be assumed that some of the excess PEI remains adsorbed in the porous structure leading to the inordinately high nitrogen content as seen in Table 1. The additional affinity of Ni to the PEI nitrogen can be assumed to assumed play a part in affecting the porous structure of the formed MRFs and this is discussed in some detail in a later section. The incorporation of Co with progressively increasing content in reaction mixture is quite representative of the expected ratios of incorporation. In the Ni3-M-RF, the experimental results show that there is only 0.7 wt% of Ni.

**Figure 1:**
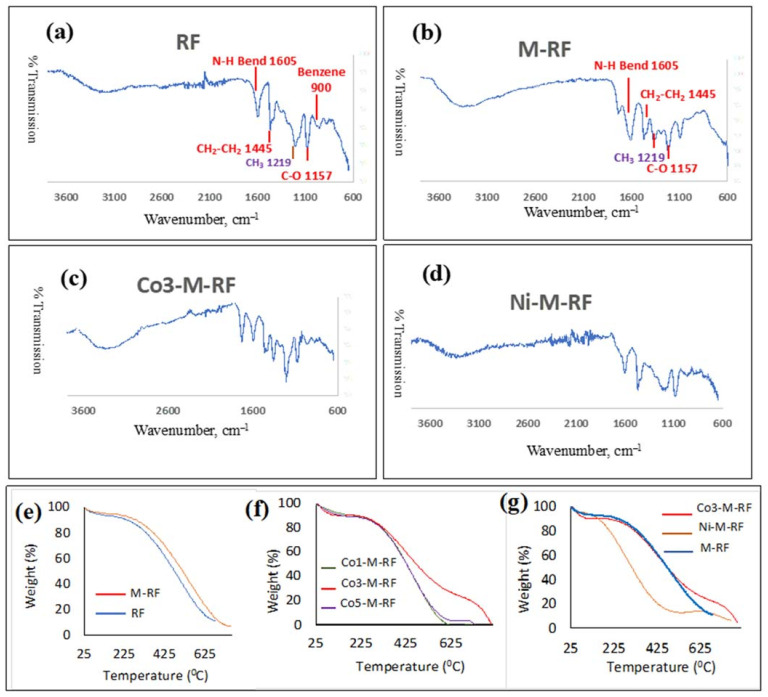
FTIR spectra (**a**) RF resin (**b**) Mesoporous catalyst without metal centers (**c**) MRF with 3%Co (Co2-M-RF) (**d**) MRF with Ni (Ni3-M-RF), Thermogravimetric Analysis (**e**) RF and MRF without metal centers (**f**) MRF with progressively increasing Co content (**g**) Comparison of MRF with MRF-3% Co and MRF with Ni.

Fourier transform infrared (FTIR) spectra was used to characterize the chemical structures of synthesized N-OMPs and these are shown in Figure 1a–d. While Figure 1a shows the spectrum of a general resorcinol formaldehyde resin, Figure 1b shows the FTIR spectra of the MRF made with 1.96% PEI. In Figure 1c (Co3-M-RF), the spectra of MRF with 1.96% PEI and 1.3%Co is shown as an example, while Figure 1d (Ni3-M-RF) shows the corresponding resin with 0.7%Ni. The change in the positions of the characteristic peaks with a change of the metal type at the center of the complex is insignificant. This points to the fact that there is very little difference in the bonding characteristic with the metal center. In the FTIR spectra, characteristic peaks associated with phenolic resins are observed. These entail absorption bands of benzene rings at 800–900 cm^−1^, the bending vibration of the C-O bond at 1157 cm^−1^, the absorption band of the methyl group at 1219 cm^−1^, the absorption bands of the methylene bridge at 1445 and 2827–2963 cm^−1^, and the stretching vibration of the O-H bond at 3200–3400 cm^−1^. Besides, the characteristic peaks associated with nitrogen species are also observed. These entail the stretching vibration of the C-N bond at 1110 cm^−1^, the bending vibration of the N-H bond at 1605 cm^−1^, and the stretching vibration of the N-H bond at 3400–3500 cm^−1^.

The thermal stability of the MRFs was evaluated via thermal analysis by heating the samples in an inert atmosphere. To remove moisture, the samples were warm up to 100 °C and then the weight loss measurement started at 50 °C to 600 °C under a constant nitrogen flow rate. The various compositions differed substantially in their thermal stability profiles and this is shown in Figure 1d–f. Thermal stability of RF is slightly better than M-RF as seen in Figure 1d. It could be construed that the presence of the amine functionality causes a more rapid breakdown of the polymeric structure with temperature due to a catalytic action. The thermal stability of 3% Co is superior to both that containing 1% and 5% Cobalt contents. The MRF with while Co3-M-RF showed more thermal stability compared to a composition containing nickel as the metal center.

### 2.2. Surface Area and Pore Structure

**Figure 2:**
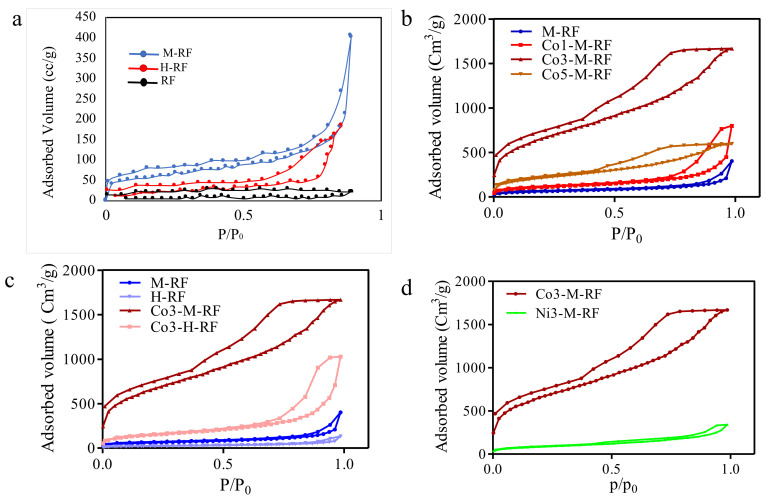
N_2_ adsorption/desorption isotherms of (**a**) RF with varying PEI concentration (0%, 1.96% and 3.98% by weight), (**b**) MRFs with varying cobalt concentrations (0%, 1%, 3% and 5% Co (III) acetylacetonate by weight, (**c**) Cobalt doped RF made with varying PEI (1.96% and 3.98%) and Cobalt concentration (0%, 3% and 5% Co(III) acetylacetonate by weight), and (**d**) Metal -doped M-RF with 3% Co (III) acetylacetonate by weight and 3% Ni(II) acetylacetonate by weight.

The nitrogen adsorption/desorption isotherms were obtained by NOVA Tech at 76 K and plotted in Figure 2. The surface area for all samples was calculated by multipoint BET model based on adsorption plot and showed in Table 2. The detailed porosity parameters of pore size volume and diameter were calculated by BJH model and are summarized in this table. Varying the concentrations of PEI, cobalt, and metal salt has a visible influence on the textural and surface morphologies of the MRFs. The porosity can be significantly improved by varying the PEI and cobalt concentrations, however the effect is not linearly correlated to their concentration.

Increasing PEI concentration, as seen in the metal-free mesoporous polymers, from 0% (RF) to a content of 1.96% (M-RF) increases the BET surface area from 19.67 m^2^/g to 325.99 m^2^/g. A further increase in the PEI content to 3.98% (H-RF) decreases it to 212.13 cm^3^/g. This is shown in Figure 2a. As per the stoichiometry of RF-PEI network formation reaction, any amount of PEI that is beyond limit of crosslinking, determined by relative component stoichiometries, will simply remain unreacted. This unreacted PEI enter the pores of the mesoporous crosslinked network in the form of a liquid and blocks them. This in turn reduces available surface area and is reflected in the BET experiment.

The results of N_2_ adsorption/desorption isotherms are shown in Figure 2a–d. With 3% Cobalt doping in the MRF containing 1.96% PEI, the BET surface area is seen to increase to 977.111 m^2^/g. This composition is designated as Co3-M-RF. With 1% Co the BET surface area decreases significantly. With a much higher 5% Co doping there is an insignificant change in surface area compared to the undoped MRF structure as well as that of Co3-M-RF. The surface area increases much faster with Cobalt doping in the MRF made with medium PEI, MRF (1.96%) than that made with higher amounts of PEI, HRF (3% doping). With M-RF, for the Co3-M-RF composition, the surface area by more than 200% over an undoped samples. While with a higher PEI loading (H-RF), the same amount of Co increases the surface area by only 61.3%. The doping using Co may be thought to induce a morphological nucleation of the polymer which, in tandem with the crosslinking kinetics forms an optimized structure with the maximum possible surface area. The nucleation and cross-linking have their own kinetic rates which appear to be experimentally optimized at 3% Co content, neither lower, nor higher percentage of Co doping see to have obtained the same optimization to maximization of surface area and this is seen in Figure 2b. In Figure 2c, the significantly higher surface area of the polymer with 3% Co and 1.96% PEI, merely underscores the points made above of the optimized level of Co and PEI incorporation to achieve the best possible results. The shape of the curves correlates very closely to type IV isotherms that are typical in mesoporous solids. The adsorption on mesoporous solids occurs vis multilayer adsorption which is followed by capillary condensation that leads to the hysteresis loop as is seen in the figures. The capillary condensation that takes place in the mesopores can somewhat limit uptake at higher partial pressures. However, in the case of RF, the obtained isotherm showed Type IV and V indicating a greater predominance of capillary condensation.

**Figure 3:**
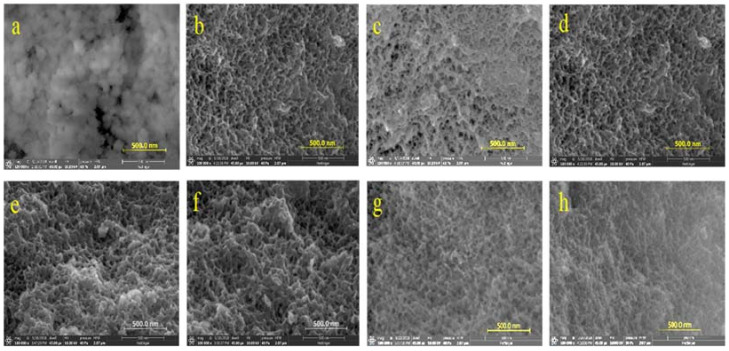
SEM of various samples: (**a**) RF (**b**) M-RF (**c**) Co1-M-RF (**d**) Co3-M-RF (**e**) Co5-M-RF(**f**) Co3-H-RF (**g**) H-RF (**h**) Ni3-RF.

The SEM images of various MRFs was illustrated in Figure 3a–h, confirmed uniform microporosity of samples contained PEI. The SEM images reflect the results and observations in the figure and table above. The spherical morphology of RF (Figure 3a) is another proof for the importance of PEI in developing a uniform morphology with an interconnected framework, which can be seen in subsequent images. The immobilization of cobalt increases pore interconnection with consequent enhancement in surface area, as mentioned above. It is obvious that excessive incorporation of either PEI or Co destroys the clean morphology that is obtained with 1.96% PEI and 3% Co, which again is testament to the empirically obtained optimization of the nucleation and crosslinking reactions. The SEM images also affirm that Ni (Figure 3h) is not as effective in inducing the morphological optimization as is seen with Co doping.

### 2.3. Carbon Dioxide Adsorption

**Figure 4:**
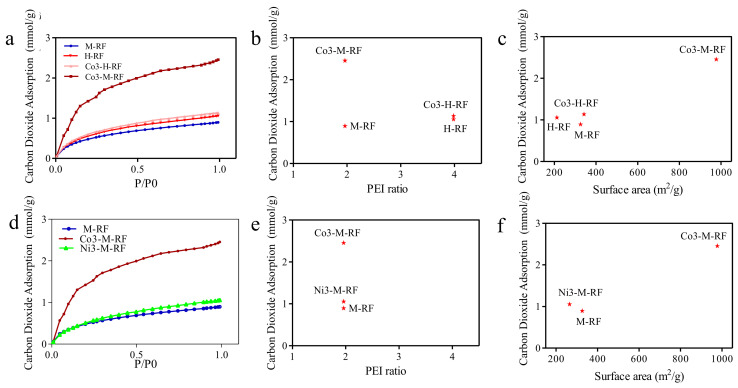
Carbon dioxide adsorption of various composition (**a**) at 0 °C, vs. P/P_0_ (**b**). CO_2_ adsorption at 0 °C vs. PEI % weight in MRF (**c**) CO_2_ adsorption at 0 °C vs. Surface area (**d**) Carbon absorption at 25 °C vs. P/P_0_ (**e**) CO_2_ adsorption at 25 °C vs. PEI % weight in MRF (**f**) CO_2_ adsorption at 25 °C vs. Surface area.

The carbon dioxide adsorption results are presented in Figure 4a–f. The MRFs were utilized as adsorbents for CO_2_ adsorption at 25 °C or 0 °C and 1 bar. CO_2_ adsorption isotherms of MRFs are shown in Figure 4a,d. The CO_2_ uptake at 1 bar is presented Figure 4b,c,e,f.

The adsorption results with Co3-M-RF shows the best adsorption results. One primary reason is that the surface areas of this sample was seen to be higher than other samples. The high PEI containing samples perhaps had quite a bit of the pores blocked by the presence of excess PEI in liquid form blocking gaseous movement through the mesopores and hence its subsequent adsorption. The is reflected in Figure 4b. A higher PEI content is expected to attract more CO_2_ due to its alkaline disposition. However, the reality remains that the compositions with higher PEI content result in lower surface area due to unreacted PEI plugging pores and hence causes diminished adsorption. The adsorption of CO_2_ is a balance between the amount of surface area proffered and the alkalinity of the surface area functionality. The importance of surface area available for adsorption is underscored in Figure 4c where the composition with the higher surface area adsorbs the higher amount of CO_2_ despite having lower content of PEI compared to H-RF structures. M-RF, which has a low surface area (325.99 m^2^·g^−1^) and pore volume (0.35 cm^3^·g^−1^), exhibits a CO_2_ uptake of 1.15 mmol·g^−1^. The Cobalt doped sample Co3-M-RF also shows a high CO_2_ adsorption capacity (2.5 mmol·g^−1^ at 0 °C and 1 bar). Ni3-M-RF displays a CO_2_ adsorption capacity of 1.05 mmol·g^−1^ at 0 °C, 1 bar. Previous research demonstrates that resorcinol-formaldehyde resin exhibit stronger Lewis basicity, and generally have good CO_2_ capture capabilities on their own. Here we see that the CO_2_ capture capability rises when a doping metal center is introduced. In Figure 4e,f, despite comparable amount of PEI, the sample with cobalt doping has superior CO_2_ adsorption because it has a much higher surface area. When the concentration of PEI is increased from 0 to 3.98%, there is very little concomitant increase in CO_2_ adsorption. However, an increase in cobalt concentration, caused the CO_2_ adsorption to be enhanced sharply. Thus, it is seen that doping causes a significant effect on nucleation and hence formation of mesopores and channels thereof, increasing the surface area, which plays a greater role in the adsorption efficiency than that proffered by the functional alkalinity obtained from higher PEI functionalization.

## 3. Discussion

Higher content of PEI in a mesoporous RF resin structure, leads to more rapid carbonization of the polymer network resulting in a sharp and rapid weight loss. Additionally, PEI is thought to attract moisture and further speed up breakdown of benzoxazine connectors with increasing temperature. This trend is reflected in the TGA results. Samples containing Cobalt as the doing center have more thermal stability compared to those containing Nickel. Nickel is known to exhibit greater bonding to PEI nitrogen atoms entailing the coordination of a donor heteroatom with an electro-deficient metal center. The lone pair of electrons on nitrogen atoms in the PEI structure form a donor complex with Nickel, causing an ensuing catalytic effect. Thus, the thermal degradation of the Nickel doped MRFs is more rapid compared to Cobalt nucleated MRFs [39,40].

Co has a more pronounced effect in increasing surface area than Ni. This is seen clearly in the polymer made with 1.96% PEI and shown in Figure 2d. While both Cobalt and Nickel have six coordination sites each, Nickel is known to have a higher affinity for amino coordination, as seen in polyhistidine tag chromatography [40]. The weaker affinity of Cobalt has been seen in protein chromatography. It results in Co being less deleterious on the structure of the protein by way of lower bonding affinity. A similar process is thought to occur in the polymers made in this work. Here, morphological nucleation works alongside and parallel to the cross-linking process in the polymeric structure. We can visualize the lone pair of electrons on the PEI amine nitrogen coordinate with the Ni vacant orbitals. This coordination complex based anchoring results in a nucleation process which competes with the rate of crosslinking. Thus, a higher affinity with Ni may cause a more pronounced disruptive effect on structure formation and porosity.

A milder coordination between PEI amine lone electron pairs and the Cobalt centers causes a more even balance between crosslinking and nucleation leading to a better development of surface area and uniform porosity [41,42]. The isotherm was found to match to Type IV adsorption curves, indicating the presence of multilayers. However, in the case of RF, the obtained isotherm showed Type IV and V.

## 4. Materials and Methods

### 4.1. Materials

Resorcinol (99%), Formaldehyde solution (37% in water), and branched polyethylenimine (MW = 10000) were purchased from Alfa Aesar (Haverhill, MA, USA) and used without any further processing. Cobalt acetylacetonate and nickel acetylacetonate (95%) were obtained from Bean Town Chemicals (Hudson, NH, USA) and used as received. Ethanol proof 200 (VWR) and sodium hydrogen sulfate were purchased from Sigma-Aldrich (St. Louis, MO, USA).

### 4.2. Synthesis of My-x-RFs

My-x-RFs were synthesized by a one-pot solvothermal method. 1.5 g resorcinol was dissolved in 4.4 mL of deionized water (DI). Then 5.6 mL of PEI (1.96% or 3.98%) *w/v* in ethanol was added, and the mixture was kept stirring at 40 °C for 20 min. Then the metal salt was added and stirred for an addition 20 min at 40 °C. Then 2.1 mL of formaldehyde solution 37% was injected by syringe quickly and the milky solution stirred at high speed for 20 min at 40 °C. Finally, this emulsion was transferred to a Teflon autoclave chamber and kept a under static condition at 120 °C for 12 h. After cooling to room temperature, the polymer was smashed and washed three times with water and then three times by ethanol. M-RFs were dried at room temperature overnight. The obtained mesoporous catalyst was named as My-x-RFs, where M is metal type, y is metal concentration in the reaction mixture, and x is the PEI concentration denoted as N (0% PEI), M (1.96% PEI) and H (3.98% PEI). RF is denoted for mesoporous resorcinol formaldehyde polymer without metal salt and PEI.

### 4.3. Characterization

Scanning electron microscope (SEM) was used to look at the morphology and pore structure of the samples. Thermo Scientific™ FEI Quanta™ line 650 SEM (Waltham, MA, USA) with a low vacuum detector under 10 kV took images. Then energy-Dispersive Spectroscopy (EDS) was used for elemental mapping analysis by FEI Quanta FEG 250 detector. Aztec software (Oxford Instruments, Abdington, UK) was applied to the analysis of EDS data. Thermogravimetric analyzer (TG) was performed on TA Instruments Q500 with 10 °C·min^−1^ to 750 °C under N_2_ with 100 m^2^/hr. To remove moisture from the samples, temperature was increased to 100 °C, and data acquisition was started after cooling down to 50 °C. The N_2_ Adsorption was measured by Quantachrome NOVA Tech LX4. Prior the measurement, the sample was degassed at 120 °C for 24 h under vacuum to remove the adsorbed species on the surface of sample and finally cooled to room temperature. The Brunauer-Emmett-Teller (BET) method was utilized to calculate the specific mesoporous surface area of the samples by using the adsorption branch acquired at a relative pressure (P/P_0_) range of 0.05–0.30 (Equation (1)). Barrett, Joyner, and Halenda (BJH) model was applied to estimate pore size volume from the quantity of N_2_ adsorbed at relative pressure (P/P_o_) of 0.99. Touch Win^TM^ software was used for all calculation which was reported in Table 1.

(1) $$\frac{1}{W\left(\left(\frac{P_{0}}{p}\right)-1\right)}=\left(\frac{1}{W_{m}C}\right)+\frac{C-1}{\left(W_{m}C\right)}\left(\frac{P}{P_{0}}\right)$$

where W is the weight of gas adsorbed at a relative pressure, P/P_0_, and W_m_ is the weight of adsorbate constituting a monolayer of surface coverage, and C “Constant”, is related to the energy of adsorption in the first adsorbed layer and consequently its value is an indication of the magnitude or strength of the adsorbent/adsorbate interactions.

Molecular structure of the samples was explored by Fourier transform infrared spectroscopy (FT-IR). IR spectra were recorded on a PerkinElmer Spectrum 100 FT-IR spectrometer.

### 4.4. Carbon Dioxide Adsorption

CO_2_ adsorption was measured by the same system (Quantachrome NOVA Tech LX4) up to 1 bar, but ice bath was used for 0 °C and water bath for room temperature experiments. The samples were degassed as mentioned above. The isosteric heat of adsorption (Qst) was calculated based on the Clausius-Clapeyron equation [43] for which the CO_2_ adsorption was conducted at two different temperatures of 273 and 298 K by Equation (2).

(2) $$\mathrm{Qst}={\mathrm{RT}}^{2}\left(\frac{\partial \mathrm{Ln}\;p}{\mathrm{Ln}\;T}\right)$$

where R is universal gas constant; T_2_ and T_1_ are two different temperatures based on Kelvin; P_2_ and P_1_ are the partial pressure at the same amount of adsorbed CO_2_ for T_2_ and T_1_.

## 5. Conclusions

In summary, this paper presented a novel, template-free and generalized method, for the fast and scalable synthesis of metal centered mesoporous polymer for CO_2_ capturing for its possible subsequent conversion by metal catalyzed electrochemical reduction. The presented method can strongly promote the industrial application of mesoporous polymer by overcoming a series of process and scale up limitation associated with the traditional template-based self-assembly route. Owing to an abundant nitrogen content, high alkaline functionality, and predominantly mesoporous nature, the synthesized MRFs demonstrated excellent capacity for CO_2_ adsorption. Based on these results, it can be concluded that a combination of metal functionality and mesoporosity is very important for CO_2_ adsorption and catalytic conversion of CO_2_. Thus, this work provides an initial novel insight for the designing porous materials with high performance for the selective capture and conversion of CO_2_ from flue gas. The FTIR results, SEM, and elemental analysis validated the incorporation of different kinds and contents of nitrogen species into the framework synthesized MRFs. The nitrogen adsorption isotherms and SEM images revealed that the synthesized MRFs have large surface areas and abundant meso–macropores. The CO_2_ and N_2_ adsorption experiments demonstrated that the synthesized MRFs have high capacity for CO_2_ at a relatively low pressure of 0.15 bar (0.64–1.47 mmol·g^−1^ at 0 °C and 0.49–0.87 mmol·g^−1^ at 25 °C). This work provides a facile approach to the targeted synthesis of nitrogen functionalized MRFs with potential applications in the s capture and electrochemical conversion of CO_2_. 

## Figures and Tables

**Table 1:** Elemental analysis of various catalyst compositions (all figures in %weight).

Sample	Carbon	Oxygen	Nitrogen	Cobalt	Nickel
RF	62.6	31.4	0	0	0
M-RF	77.6	20.1	2.3	0	0
H-RF	60.7	28.9	10.4	0	0
Co1-M-RF	60.9	31.3	7.4	0.4	0
Co3-M-RF	51.9	37.8	9.0	1.3	0
Ni3-M-RF	73.2	23.4	2.7	0	0.7
Co5-M-RF	54.67	37.12	5.42	2.79	0
Co3-H-RF	63.9	33.3	2.0	0.8	0

**Table 2:** Surface area and pore size distributions of various catalyst based on BJH model. (Standard deviation was within (±3–4%) of the average values).

Sample	BET Data	Mesoporous Based on BJH Model		
	Surface Area (m^2^/g)	Surface Area (m^2^/g)	Pore Volume (cm^3^/g)	Pore Size Radius (nm)
RF	19.67	13.53	0.041	
M-RF	325.99	207.01	0.35	1.62
H-RF	212.13	80.07	0.34	2.39
Co1-M-RF	220.12	108.11	1.14	1.99
Co3-RF	149.80	149.80	0.25	1.89
Co3-M-RF	977.11	226.09	1.83	
Co3-H-RF	342.22	123.29	0.15	
Co5-M-RF	375.19	98.73	0.71	1.53
Ni3-M-RF	264.82	87.08	0.20	1.69

## Data Availability

Data is contained within the article or supplementary material.

## Supplementary Materials

The following are available online, Figure S1: EDS elemental analysis of various samples: (**a**) RF (**b**) M-RF (**c**) H-RF, Figure S2: EDS elemental analysis of various samples: (**d**) Co1-M-RF (**e**) Co3-M-RF (**f**) Co5-M-RF (**g**) Ni3-M-RF.

## Sample Availability

Samples of the compounds Co1-M-RF, Co3-M-RF, Co5-M-RF, Ni3-M-RF are available from the authors.
