# Supplementary material for: Developing Eco-Friendly and Cost-Effective Porous Adsorbent for Carbon Dioxide Capture
Source: Molecules. 2021 Mar 31;26(7):1962. doi: 10.3390/molecules26071962 (PMC8037370; doi:10.3390/molecules26071962)
Supplement: Supplementary file 1 [file molecules-26-01962-s001.pdf]

molecules

Supplementary Materials

Developing Eco-friendly and Cost-effective Porous Adsorbent for Carbon Dioxide Capture

Mahboubeh Nabavinia [1], Baishali Kanjilal [2], Noahiro Fujinuma [1], Amos Mugweru [3] and Iman Noshadi [1,2,*]

[1] Department of Chemical Engineering, Rowan university, Glassboro, NJ, 08028, USA, nabavinim1@students.rowan.edu (M.N.); fujinuma@rowan.edu (N.F.)
[2] Department of Bioengineering, University of California, Riverside, Riverside, California, 92516, USA; baishali.kanjilal@gmail.com
[3] Department of Chemistry, Rowan university, Glassboro, NJ, 08028, USA; mugweru@rowan.edu
* Correspondence: imann@ucr.edu; Tel.: +01-856-818-6102

Citation: Lastname, F.; Lastname, F.; Lastname, F. Title. *Molecules* **2021**, *26*, x. https://doi.org/10.3390/ molecules26071962

Academic Editor: Maurizio Peruzzini
Received: 3 March 2021
Accepted: 20 March 2021
Published: 31 March 2021

Publisher's Note: MDPI stays neutral with regard to jurisdictional claims in published maps and institutional affiliations.

Copyright: © 2021 by the authors. Licensee MDPI, Basel, Switzerland. This article is an open access article distributed under the terms and conditions of the Creative Commons Attribution (CC BY) license (http://creativecommons.org/licenses /by/4.0/).

Abstract: To address the issue of global warming and climate change issues, recent research efforts have highlighted opportunities for capturing and electrochemically converting carbon dioxide (CO_2). Despite metal doped polymers receiving widespread attention in this respect, the structures hitherto reported lack in ease of synthesis with scale up feasibility. In this study, a series of mesoporous metal-doped polymers (MRFs) with tunable metal functionality and hierarchical porosity were successfully synthesized using a one-step copolymerization of resorcinol and formaldehyde with Polyethyleneimine (PEI) under solvothermal conditions. The effect of PEI and metal doping concentrations were observed on physical properties and adsorption results. The results confirmed the role of PEI on the mesoporosity of the polymer networks and high surface area in addition to enhanced CO_2 capture capacity. The resulting Cobalt doped material shows excellent thermal stability and promising CO_2 capture performance, with equilibrium adsorption of 2.3 mmol CO_2/g at 0°C and 1 bar for at a surface area 675.62 m^2/g. This mesoporous polymer, with its ease of synthesis is a promising candidate for promising for CO_2 capture and possible subsequent electrochemical conversion.

Keywords: carbon dioxide; capture; mesoporous polymer; cobalt; nickel

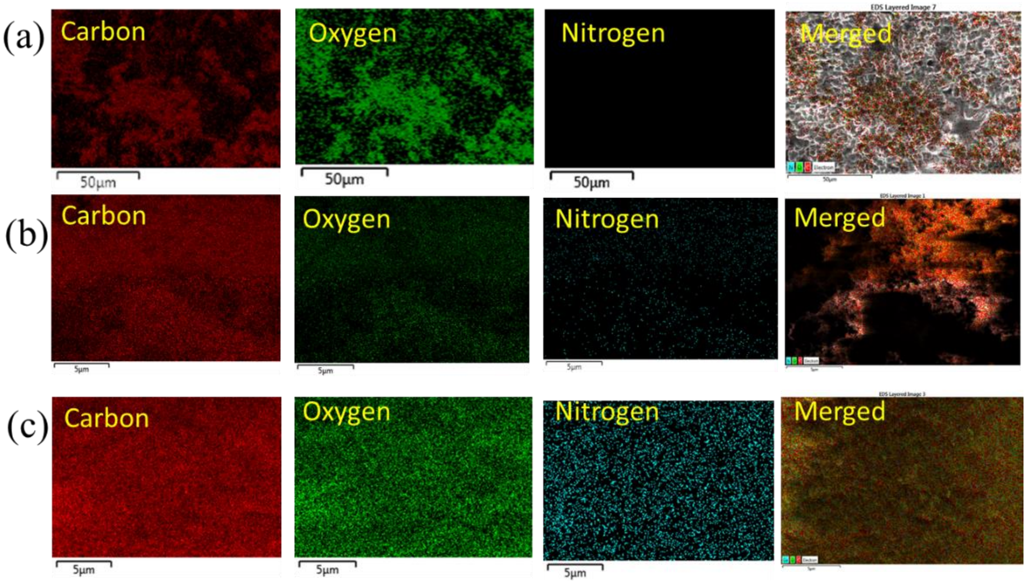

Figure S1. EDS elemental analysis of various samples: (a) RF (b) M-RF (c) H-RF.

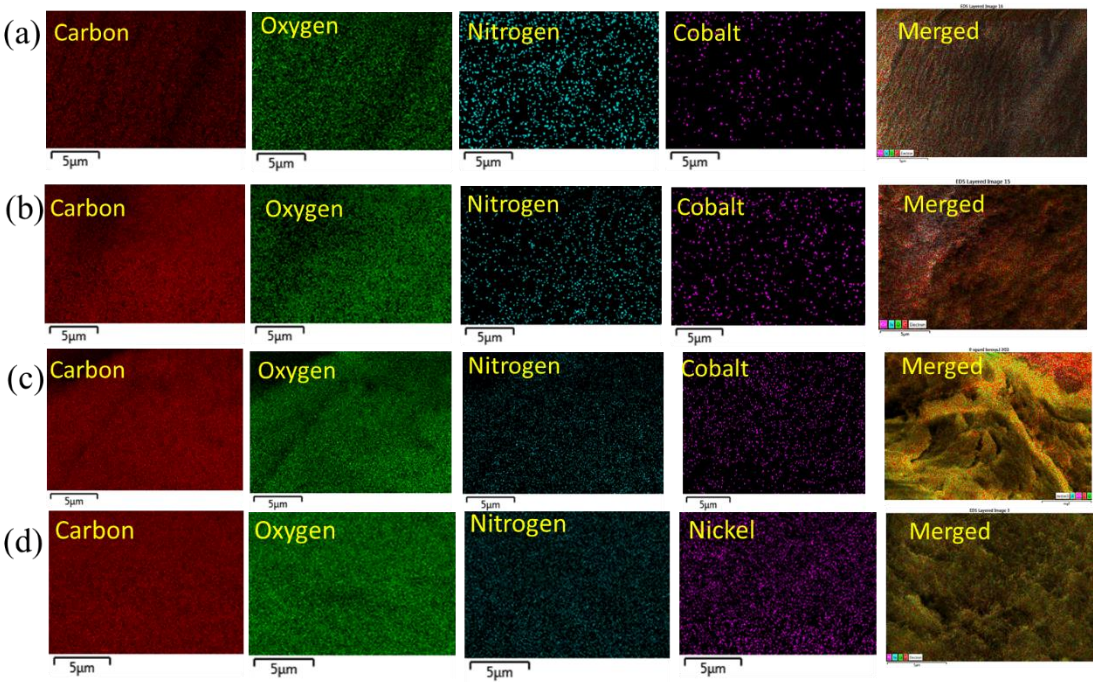

Figure S2. EDS elemental analysis of various samples: (a) Co1-M-RF (b) Co3-M-RF (c) Co5-M-RF (d) Ni3-M-RF.
